# Autologous Platelet and Extracellular Vesicle-Rich Plasma as Therapeutic Fluid: A Review

**DOI:** 10.3390/ijms24043420

**Published:** 2023-02-08

**Authors:** Kaja Troha, Domen Vozel, Matevž Arko, Apolonija Bedina Zavec, Drago Dolinar, Matej Hočevar, Zala Jan, Matic Kisovec, Boštjan Kocjančič, Ljubiša Pađen, Manca Pajnič, Samo Penič, Anna Romolo, Neža Repar, Vesna Spasovski, Nejc Steiner, Vid Šuštar, Aleš Iglič, Damjana Drobne, Ksenija Kogej, Saba Battelino, Veronika Kralj-Iglič

**Affiliations:** 1 Department of Otorhinolaryngology and Cervicofacial Surgery, University Medical Centre Ljubljana, SI-1000 Ljubljana, Slovenia; 2 University of Ljubljana, Faculty of Medicine, SI-1000 Ljubljana, Slovenia; 3 University of Ljubljana, Laboratory of Clinical Biophysics, Faculty of Health Sciences, SI-1000 Ljubljana, Slovenia; 4 Department of Molecular Biology and Nanobiotechnology, National Institute of Chemistry, SI-1000 Ljubjana, Slovenia; 5 Department of Orthopedic Surgery, University Medical Centre, Zaloška 9, SI-1000 Ljubljana, Slovenia; 6 MD-RI Institute for Materials Research in Medicine, Bohoričeva 5, SI-1000 Ljubljana, Slovenia; 7 Department of Physics and Chemistry of Materials, Institute of Metals and Technology, SI-1000 Ljubljana, Slovenia; 8 University of Ljubljana, Laboratory of Physics, Faculty of Electrical Engineering, SI-1000 Ljubljana, Slovenia; 9 University of Ljubljana, Research Group for Nanobiology and Nanotoxicology, Biotechnical Faculty, SI-1000 Ljubljana, Slovenia; 10 Institute of Molecular Genetics and Genetic Engineering, University of Belgrade, 11000 Belgrade, Serbia; 11 University of Ljubljana, Laboratory of Clinical Biophysics, Faculty of Medicine, SI-1000 Ljubljana, Slovenia; 12 University of Ljubljana, Chair of Physical Chemistry, Faculty of Chemistry and Chemical Technology, SI-1000 Ljubljana, Slovenia

**Keywords:** platelet-rich plasma, platelets, centrifugation, extracellular vesicles, small cellular particles, wound healing, regeneration, fresh-frozen plasma, intercellular signaling peptides and proteins

## Abstract

The preparation of autologous platelet and extracellular vesicle-rich plasma (PVRP) has been explored in many medical fields with the aim to benefit from its healing potential. In parallel, efforts are being invested to understand the function and dynamics of PVRP that is complex in its composition and interactions. Some clinical evidence reveals beneficial effects of PVRP, while some report that there were no effects. To optimize the preparation methods, functions and mechanisms of PVRP, its constituents should be better understood. With the intention to promote further studies of autologous therapeutic PVRP, we performed a review on some topics regarding PVRP composition, harvesting, assessment and preservation, and also on clinical experience following PVRP application in humans and animals. Besides the acknowledged actions of platelets, leukocytes and different molecules, we focus on extracellular vesicles that were found abundant in PVRP.

## 1. Introduction

Plasma is a yellowish liquid that presents a platform for blood cells, nanoparticles and molecules circulating throughout the organism. Larger particles in blood (e.g., blood cells) can be relatively effectively separated from the rest of the blood components by centrifugation. Centrifugation velocity depends on the size and density of the particles, and allows for distinction of the cell types. With proper processing, erythrocytes (and other cells of similar size, e.g., leukocytes) can be separated from the rest of blood constituents to yield plasma rich with smaller particles (platelets, extracellular vesicles, lipoproteins, antibodies, etc.). Such material was found to have healing properties [1] which were initially attributed to platelets; it was found that activated platelets release different growth and inflammation factors to the extracellular milieu, and these have been regarded as vectors of the healing process [2]. With time, other features were considered, e.g., the effects of platelet membrane receptors and of leukocytes on the immunomodulatory actions of the innate and adaptive immune system (reviewed in Everts et al.) [3], indicating complex interactions between various cells in the healing process.

Recent developments in the field of biology and medicine have outlined nano-sized (ranging from 20 nm–1000 nm) membrane-enclosed cellular fragments (extracellular vesicles (EVs)) in preparations from blood [4,5,6,7,8]. Moreover, EVs have been found in practically all biological fluids with important roles in transporting membrane proteins, cytosolic proteins and nucleic acids [9]. This has opened new areas of research in the pathophysiology of different diseases and indicated the development of new diagnostic and therapeutic possibilities [10]. In order to emphasize platelets and EVs in processed blood, the term “platelet and extracellular vesicle-rich plasma” (PVRP) has been suggested [11] to refer to a fraction of blood devoid of erythrocytes and containing increased platelet and EV concentrations with respect to blood.

EVs are continuously shed from the cells and can travel with circulation to reach non-local environment [9]. As they are enclosed by a phospholipid membrane, their cargo deriving from the origin cell is protected from lysing enzymes as long as they keep their integrity. This mechanism indicates a possibility of efficient delivery of EV content and thereby interaction between cells connected by the circulation.

Plasma processing causes an increase in platelet concentration above its baseline level in whole blood [8,12]. Autologous plasma is prepared from the patient’s own blood following procedures that are straightforward and easy to perform: the patient’s peripheral blood is collected into anticoagulant-containing test tubes and fractionated, most commonly, in a two-step centrifugation process. In the first step, blood is centrifuged to separate erythrocytes (the centrifugation pellet) from the rest of the blood. In the second step, plasma that forms the supernatant of the first step is centrifuged again to concentrate platelets in the bottom of the centrifugation tube. The concentrated part that constitutes PVRP is ready to be applied directly to the targeted area, where it undergoes endogenous activation. It can be exogenously activated by the addition of calcium mixtures prior to the application onto tissues yielding a gel-like preparation (PVRP gel) [13]. In this way, the healing substances are delivered to places where blood would otherwise rarely go on its own. With autologous application there is low risk of complications, such as infections or immune rejection [14]. Its counterpart, allogeneic PVRP, is much less extensively studied, most notably due to its immunogenicity, infection risk and the need for time-consuming donor selection. However, it may be useful in patients, who are unfit for large blood quantity harvesting, such as in severe traumatic conditions, acute burns with fluid depletion or thrombocytopenia, sepsis, hematological disorders, infections, in the elderly, in neonates or in patients with relative contraindications (corticosteroids, anemia, malignancies, etc.) [15,16].

Autologous plasma as a therapeutic liquid is presently being considered in different fields of medicine and veterinary medicine. However, due to its complex composition and physical properties, and due to its dynamic nature, the knowledge on the processes taking place in plasma is currently rudimentary. Furthermore, the contents of PVRP depend on the processing methods. Some protocols are focused on the content of leukocytes connected with the concentration of specific molecules [17]. The protocols emphasize the importance of adequate balance of catabolic and anabolic processes in regard of specific pathophysiologic conditions [18]. The key questions regarding autologous plasma are which and in what context the components of plasma have beneficial effects on particular mechanisms.

In this review, we focus on clinical aspects that may be connected to plasma contents. In this light, we will describe the composition of PVRP, the methods for preparation of PVRP, the methods of characterization of PVRP and clinical experiences with PVRP in human and veterinary medicine.

## 2. Composition of Plasma Rich with Platelets and Extracellular Vesicles 

Plasma is composed of water and plasmatic proteins (the most abundant of which are albumins), small extracellular particles, dissolved molecules and ions [19,20]. Plasma serves as a medium of blood cells—erythrocytes, leukocytes and platelets. PVRP has various compositions depending on the preparation procedure, and donor’s physiological or pathophysiological condition [21]. The main biological contents of PVRP are discussed below.

### 2.1. Platelets

Platelets are the second most prevalent blood component after erythrocytes [22], sized 2–5 µm. A mature platelet does not contain a nucleus. Platelets are formed in bone marrow or lungs by fragmentation of a megakaryocyte and circulate in the bloodstream for 7–10 days [23]. In the bloodstream, they interact with blood constituents, in particular with endothelial, immune and other circulating cells. By responding to the changes in their surface properties they play important roles in hemostasis, thrombosis, immune system activation and tissue regeneration [24].

From a historical perspective, following descriptive studies, the platelet research turned to molecular mechanisms, including single molecule biophysics, single cell biology, single cell molecular biology, structural biology, computational simulations and high-throughput data-dense techniques [25]. The current research is focused on the “nanostructurome”, which we expect to become filled with data on nano-sized particles derived from platelets.

**Figure 1:**
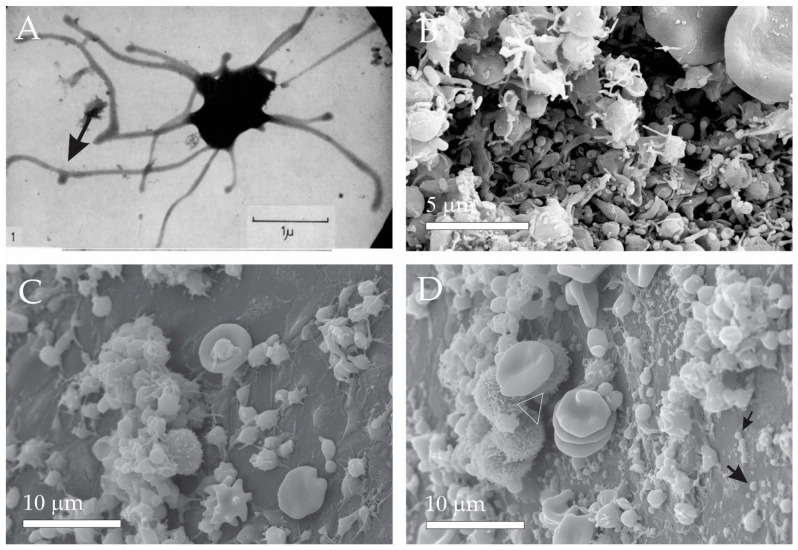
Micro and nano-sized plasma constituents. (**A**) Transmission Electron Micrograph (TEM) of an activated platelet showing budding of the tubular protrusion. (**B**) Scanning Electron Micrograph (SEM) of erythrocytes (parts shown in upper right corner), activated platelets and numerous EVs. (**C**) SEM of plasma after 15 h fasting. (**D**) SEM of plasma after a greasy meal revealing numerous lipid droplets (black arrows) and a few leukocytes (white triangle). (**A**): From Wolf, 1967, reprinted with permission from the British Journal of Haematology [46]. 2008, John Wiley and Sons (license number: 5458180157633). The sample shown in Panel (**B**) was prepared as described in [4]. The samples shown in Panel (**C**,**D**) were prepared as described in [47].

The proposed mechanism of platelets in preventing blood loss at sites of injury is well-acknowledged: platelets are considered to adhere, aggregate and form a procoagulant surface, resulting in thrombin and fibrin [19,26]. The role of platelets releasing substances from their storage pools and their role in tissue repair, angiogenesis and inflammation, however, has been further studied in recent decades [27]. Growth factors, cytokines, chemokines and newly synthesized active metabolites have been shown to be contained in platelets completing their non-hemostatic roles, such as the activation of fibroblasts, recruitment of leukocytes to the injury site and inducing cell proliferation as well as the migration of other cells associated with tissue healing (see Figure 1) [28].

Compared to the baseline serum levels, the number density of platelets in PVRP reaches an approximately 2.8-fold increase [8,11]. The optimal concentration of platelets to elicit a therapeutic effect has been a matter of debate, a definite consensus on adequate concentration is lacking. While the number densities of (0.2–1) × 10^6^/mL have proved to have a therapeutic effect [29], higher number densities have been found unbeneficial [30]. In fact, very high growth factor concentrations released from numerous platelets have been found to lessen the sensitivity and expression of their receptors, possibly eliciting unwanted effects [27,30,31].

### 2.2. Leukocytes

Leukocytes represent less than 1% of whole blood volume with the concentration of 4–10 × 10^9^/L in peripheral blood. They exert their functions by being transported by blood to their target sites. Based on the specific granules in cytoplasm, they are categorized into granulocytes (neutrophiles, eosinophiles, basophiles) and agranulocytes. The main function of neutrophiles is the phagocytosis of bacteria and dead cells. Moreover, they are the key mediators in the early phase of the acute inflammatory reaction in response to tissue injury. The non-granulated leukocytes are further categorized into lymphocytes and monocytes, which have the ability to morph into other active cells and are also involved in phagocytosis and the immune response [19].

Leukocytes play important roles in plasma preparations, affecting the overall clinical outcome [18]. Several studies examined the effect of leukocyte-rich PVRP concluding that preparations with high leukocyte contents predominantly act in catabolic and inflammatory manner by increasing pro-inflammatory cytokines and matrix metalloproteinase [18,32,33]. A recent meta-analysis of 32 studies examining the effects of leukocyte-poor PVRP compared to leukocyte-rich PVRP in knee osteoarthritis patients showed that intraarticular PVRP injections resulted in significant pain improvement regardless of leukocyte concentration. The study, however, showed increased reports of adverse reaction, such as pain and swelling after PVRP injection, depending on leukocyte concentration [34]. Zhou et al. (2015) suggested that L-PVRP should not be used in treatment of chronic tendon injuries which are associated with chronic inflammation and degeneration, as this could result in delayed healing [18].

On the other hand, leukocytes are a crucial link in the inflammatory phase of healing by increasing cell proliferation for tissue remodeling and tissue contraction [18,35]. Additionally, leukocyte contents have been found effective in eliminating microbes by activating macrophages, which migrate to the wound site after neutrophil secretion of chemoattractant cytokines [36]. Lana et al. found leukocytes to be more beneficial than detrimental in the process of healing, necessary for “regenerative inflammation” [37]. It has also been suggested that the L-PVRP could be useful in acute tendon injuries with desired proinflammatory action to diminish scar formation [38]. Thus, depending on the healing stage and type of injury, leukocytes may be a much needed source of regeneration molecules provided by PVRP [37].

### 2.3. Molecules

Platelets contain more than 1100 different protein types with numerous post-translational modifications, resulting in more than 1500 protein-based bioactive factors [39]. The most abundant proteins in platelets are signaling proteins, including growth factors (epidermal growth factor (EGF), vascular endothelial growth factor (VEGF), transforming growth factor-beta (TGF-β), insulin-like growth factor-1 (IGF-1), chemokines and other cytokines (interleukin-1β, platelet basic protein, platelet factor 4, C–C chemokine ligand 5), adhesion proteins (vitamin D-binding protein, plasminogen, fibrinogen, fibronectin, vitronectin), proteases and antiproteases [40]. The origin of some molecules deriving from platelets (Table 1) was categorized into three types of granules: α-granules, dense or δ-granules and lysosomes [11,41,42]. α-granules were found to be the most abundant and largest; dense or δ granules were found less abundant, smaller and containing smaller molecules (e.g., ADP, serotonin, polyphosphates, glutamate and histamine); lysosomes were found the least abundant and containing different enzymes, such as glycohydrolases and others that degrade glycoproteins, glycolipids and glycosaminoglycans [42]. Platelets also contain various forms of RNA, in free form or enclosed in vesicles. Recent studies have indicated that platelets may have synthetic capabilities [43].

PVRP has been shown to contain amino acids, hormones (insulin, estradiol, adrenocorticotropic hormone, androgens, estrogens, progesterone and human growth hormone), corticosteroids, thyroxine, serotonin, epinephrine, histamines, enzymes, vitamins, organic acids, pigments, ions, dissolved gases, nutrient molecules and metabolites [44,45].

### 2.4. Extracellular Vesicles

It was observed that blood products such as plasma and serum contain material in minute particulate form, sedimentable by high-speed centrifugation and originating from platelets, but distinguishable from intact platelets [46]. It was suggested that this material, then named ‘platelet-dust’, is responsible for the phenomena such as the generation of thrombin on recalcification of plasma freed from intact platelets, and platelet-like activity in serum [46]. Furthermore, it was suggested and supported by images that the particles (i.e., extracellular vesicles (EVs)) are shed off from activated platelets (Figure 1A, black arrow). Figure 1B shows plasma constituents: activated platelets, erythrocytes (partly seen in the upper right corner) and numerous EVs, heterogeneous in size and shape. Figure 1C,D show plasma after 15 h of fasting and after a greasy meal, respectively. It can be seen that after a greasy meal, plasma is enriched in small particles, most likely lipoproteins. Small particles in Panels B and D are different in shape: the ones in Panel B correspond to the minimal membrane free energy, indicating that they are membrane-bound liquid. On the other hand, the droplets have irregular globular shapes.

It is now acknowledged that besides in coagulation, EVs play a role in cellular signaling, vascular injury and homeostasis [48]. EVs exert the effects of their contents by interacting with the target cell membrane. Target cells can either be distant cells, neighboring cells or the origin cell itself. Three mechanisms of action on target cells have been described: EVs can produce effects by binding to the membrane receptors, fusing with the plasma membrane and unloading the contents into the lumen of the target cell or by endocytosis into the cell [49,50]. The secretion of EVs seems to be highly conserved throughout evolution, as it has been found that all cell types, eukaryotic and prokaryotic cells, are able to secrete EVs both in vivo and in vitro [51]. In pluricellular organisms, EVs have been found in many body fluids and tissues; blood, urine, saliva, breast milk, amniotic fluid, ascites, cerebrospinal fluid, bile, semen, cartilage and tumor tissues [52]. Over the years, designated protocols for EV isolation from body fluids and cell cultures have been developed [53].

Recent discoveries indicate that EVs mediate communication between cells by transporting cargos such as membrane proteins, cytosolic proteins, lipids and nucleic acids (e.g., DNA, messenger RNA (mRNA), microRNA (miRNA), long non-coding RNA (lncRNA) and circular RNA (circRNA)) to more or less distant cells. The mechanisms of the cargo loading and unloading are selective, depending on the cell type and conditions of the tissue [6,49]. By internalization into recipient cells, EVs may regulate various signaling pathways and influence physiological and pathological states of the receiving cell [54].

Attempts have been made to classify EVs based on their origin, size, morphology and cargo content into microvesicles and exosomes [49,55]. Microvesicles were defined as EVs (ranged 100–1000 nm) budding from the plasma membrane and exosomes were defined as EVs (ranged below 150 nm) formed in endosomes which are released upon fusion of endosomes with the plasma membrane into the extracellular space. Later, apoptotic bodies have been added to the classification, as EVs (50–1000 nm in diameter) released from cells undergoing apoptosis [49,56,57]. As it is becoming acknowledged that EVs may be created or transformed during the processing of samples [4] where observation of EVs is only possible, the origin of the observed EVs remains obscure. 

Depending on the cell type of EV origin, the particles may display specific proteins, involved in different roles and functions [57]. However, due to their small size and fragility, EVs cannot be observed directly in their native environment. The samples should undergo processing, which may transform or destroy EVs. Once in the isolate, the origin of the particles is obscured. Therefore, the latest recommendations of the International Society of Extracellular Vesicles (ISEV) suggest that when the origin of the particles is not particularly clear, the above classification should rather be replaced by the terms extracellular vesicles (EVs) and extracellular particles (EPs) [53].

**Figure 2:**
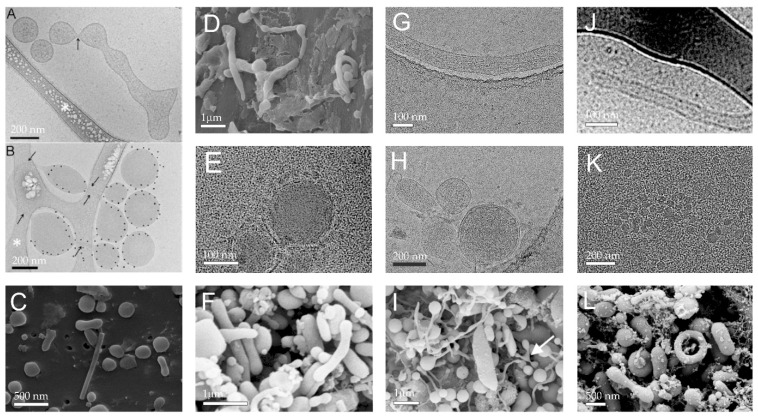
Selected images of EVs found in isolates from plasma. (**A**,**B**) Cryogenic Transmission Electron Micrographs (cryo-TEM) of EVs labeled with Anx5-, anti-CD235a- and anti-CD41-gold-NPs. White asterisks in (**A**,**B**) point to areas of the carbon net. Black arrows point to thin necks formed within the vesicles. Scale bars: 200 nm. (**C**,**D**,**F**,**I**,**L**): SEM of isolates from human plasma. (**E**,**G**,**H**,**J**,**K**): Cryo-TEM of isolates from plasma. (**A**,**B**), (**E**,**J**): Reproduced with permission from John Wiley and Sons, published by Journal of Extracellular Vesicles, 2013 (license number: 54583331312062) [60]. (**D**) From [10]. (**I**) Originally published by and used with permission from Dove Medical Press Ltd. [4]. Samples depicted in Panels (**C**,**G**,**H**,**K**) were prepared as described in [11]; samples (**F**,**L**) were prepared as described in [4].

EVs have been imaged by electronic microscopy. Figure 2 shows selected images of EVs found in isolates from plasma. In accordance with the structures observed in plasma, the isolated EVs attain globular and tubular shapes. As they are a dynamic system, their shape changes subject to external impacts and redistribution of material within the membrane takes place. By observing EVs with electronic microscope techniques, it has been concluded that EVs in blood are a dynamic material from fragmented blood cells and other blood contents [4,7,58]. Figure 2A shows transformation from a tubular shape into undulated tubular shape and finally fragmentation into globular vesicles, as observed by cryo-TEM (cryogenic electron microscopy). It was speculated that the fragmentation resulted from mechanical constraints associated with shear effects during thin film formation [59]. The sample was labeled with Anx5-, anti-CD235a- and anti-CD41-gold-NPs that were supposed to attach to the receptors on erythrocytes, but these particles cannot be seen in Figure 2A. Figure 2B shows the gold particles, indicating that the particles in Figure 2B are of erythrocyte origin. Figure 2E shows Cryo-TEM of globular membrane-enclosed EVs surrounded by smaller spherical particles that can be seen also in Figure 2K. The bilayered membrane cannot be resolved in these particles. Figure 2A,D,G,I present tubular EVs and Figure 2A–C,E,F,H,I,K,L present globular EVs found in the isolates. Figure 2I shows isolate from mare plasma and all other panels are of isolates from human plasma. Panel I reveals a mode of fragmentation of material (white arrow), in line with the process shown in Panel A. Some EVs attain peculiar shapes; a torus can be observed in Figure 2L. All blood donors were without record of disease.

### 2.5. Influence of Different Physiological and Pathophysiological Conditions on Plasma Rich with Platelets and Extracellular Vesicles 

It was found that blood contents reflect the conditions of the body. In this aspect, it has been suggested that the high variability of PVRP treatment outcomes is not only due to different equipment and protocols but also due to the difference in patients’ plasma composition [61,62]. Even though the majority of available studies on PVRP characteristics are based on blood obtained from healthy volunteers, several inherent factors affecting the PVRP composition and treatment outcomes have been proposed. Significant differences in PVRP constituents have been observed at different ages and sex of the examined groups, with higher levels of particular cytokine and growth factors present in younger subjects and in males [45,62]. Additionally, PVRP content was investigated in obese individuals, who are known to generally exert higher levers of chronic inflammation and circulating pro-inflammatory cytokines. Nevertheless, diet has been shown to affect plasma concentrations as seen in Figure 1C,D, depicting different plasma composition after 15 h of fasting and after a greasy meal (unpublished data).

The feasibility of PVRP products has additionally been explored in inflammatory and trauma states [63], which are common settings for PVRP applications. It has been acknowledged that after trauma, the activation of the immune system raises the level of proinflammatory cytokines, elevates leukocyte count and alters platelet concentrations [64]. The patient’s age, sex and physical activity have been shown to affect granulocyte and lymphocyte levels, which are crucial in facilitating fracture and wound healing [65,66]. Verboket et al. investigated the influence of trauma and surgery on the cellular and humoral composition of platelet-rich fibrin product and reported significantly elevated percentage of inflammatory monocytes and higher proinflammatory (IL-6) and anti-inflammatory (IL-10) cytokines in injured patients. Moreover, the researchers demonstrated the influence on the products by the systemic inflammation by activated immune cells, platelets and humoral mediators. This was observed by the visible leukocyte contents within the histological samples of the platelet-rich fibrin clots, which correlated with the peripheral blood leukocyte concentration, while the dimension of the clot inversely correlated to the platelet concentration in blood [67]. Single nucleotide polymorphisms were also investigated as possible factors for various responses of patients in the tissue healing process. Researchers Szyluk et al. demonstrated that polymorphic variants of PDGFRB gene, which encodes the PDGFRB receptor involved in the development of blood vessels and in the bone healing process, influence the effectiveness of PVRP treatment of tennis elbow [68].

The complexity of interactions influencing PVRP composition-related specific host characteristics emphasize the importance of individual consideration of PVRP products in clinical use [12,69].

## 3. Preparation of Platelet and Extracellular Vesicle-Rich Plasma

The fractionation of blood components in PVRP preparation is typically performed by centrifugation, where the physical force from continuous revolutions pushes denser and larger particles to the outer edge of the sample, forming roughly three layers: erythrocytes at the bottom, a mixture of leukocytes and platelets (called the buffy coat) in the middle and a layer of plasma at the top [70]. The preparation of PVRP can be done in a relatively simple procedure in various settings. For therapeutic preparations, the processing can be performed immediately after blood harvesting in a laboratory, operating room or outpatient clinic. Depending on the method, blood withdrawal volume can be estimated based on platelet yield [11,69]. The volume of withdrawn blood depends on the requirement to cover the targeted area. A range of 8–120 mL of withdrawn blood was reported to be obtained for wound care applications, skin rejuvenation and hair rejuvenation; 50–60 mL of peripheral blood is usually obtained for orthopedic interventions; 30–55 mL is obtained for standard chronic wound care [71,72,73]. Withdrawn blood needs to be anticoagulated to prevent platelets to coalesce into a clot and disable the release of bioactive molecules. This is usually performed by blood withdrawal into sodium citrate tubes [69].

Two centrifugation steps can be applied in plasma preparation: the first centrifugation step divides erythrocytes and leukocytes from plasma containing platelets, while the second centrifugation step concentrates the platelets [12,74]. An important factor in PVRP preparation is the platelet activation mode. In the PVRP preparation procedure, the platelet activation can be performed endogenously, allowing for the activation to occur when plasma is applied on the biological surface, or exogenously prior to the application on tissues. In the tissues, platelet activation is induced by collagen type I. Endogenous activation of platelet-products acts somewhat more physiologically, in a slow but potent manner [75]. On the other hand, the exogenous addition of thrombin causes a quick surge in growth factor release, diminishing available concentrations of growth factors and other bioactive molecules very quickly in the process. Thrombin acts through receptor-specific platelet activation signaling pathways which induce changes in platelet shape, secretion of granules and “inside-out” signaling process [76]. Moreover, the addition of thrombin to plasma was reported to induce the formation of fibrin fibers and a three-dimensional network consisting from these fibers [77] that contributes to the form of PVRP gel. Reactions to bovine thrombin have been reported by formation of antibodies against the substance, potentially leading to a rare but serious immune-mediated coagulopathy [30]. Calcium chloride allows for a somewhat slower release of growth factors from the preparation [29,60].

**Figure 3:**
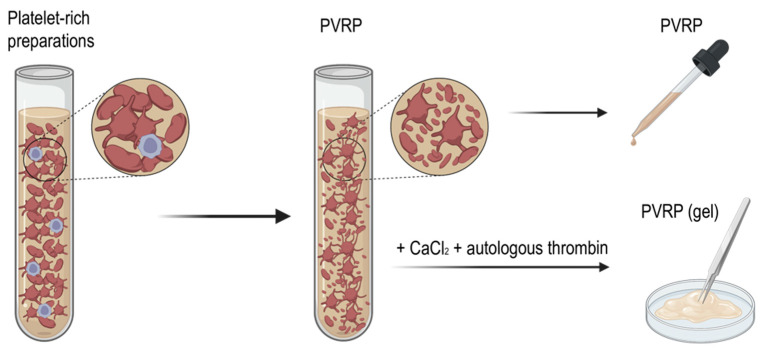
A schematic display of PVRP and PVRP gel preparation. PVRP, platelet and vesicle-rich plasma. Source: Biorender.com.

The type of activation defines the form of the product (a liquid solution or a gel). By activating the preparation exogenously, a gel-like consistency of the preparation is formed, due to clot formation [30]. This preparation is referred to as PVRP gel (Figure 3). To prepare PVP gel, calcium chloride and autologous serum is used for exogenous platelet activation [69]. PVRP gel enables easier manual application of localized, undispersed spreading onto the tissues. Štukelj et al. have emphasized that platelets are activated already during centrifugation and during the application of the preparation [7].

Apart from the described preparation technique, the patient’s blood characteristics determine the PVRP contents and its effects [7]. Andia et al. emphasized optimization of platelet-rich products regarding the desired effect on target tissues by identifying the critical molecules and designing formulations based on the disease characteristics [78]. Three main technical features have been outlined to influence the components of these products and can be modified during the preparation: the cellular composition (containing leukocytes or not), the proportion of pro- and anti-inflammatory molecules and method of product activation—depending on the need of acute or gradual for of bioactive substance release. In this aspect, several strategies are being investigated, selecting specific cytokines in platelet cargo [44]. The protocol for plasma preparation can also be supported by mathematical modeling to optimize the platelet and EV content of plasma of an individual [12]. Such an approach has already been applied in clinical practice [69].

The absence of standardized procedures, however, has led to the introduction of a variety of different platelet-rich bioformulations into clinical practice [3,21]. Descriptions of biomaterials with different additives, composition (with or without fibrin, leukocytes, red blood cells), formulations (liquid, solid, gel) and modes of application (injected, topically applied) are found in the literature [3,79]. Table 2 includes the most common platelet-rich products, their preparations and indications, including the more recently developed second-generation fibrin-based products. To emphasize the presence of EVs in all these products, we have abbreviated the products as follows: P-PVRP, Platelet and Extracellular Vesicles-Rich Plasma; LP-PVRP, Leukocyte-Poor Platelet and Extracellular Vesicles-Rich Plasma; L-PVRP, Leukocyte, Platelet and Extracellular Vesicles-Rich Plasma; P-PVRF; Pure-Platelet and Extracellular Vesicles-Rich Fibrin or Leukocyte-Poor Fibrin; L-PVRF, Leukocyte, Platelet and Extracellular Vesicles-rich Fibrin [15,80,81,82].

## 4. Characterization of PVRP Composition and Methodological Approaches

### 4.1. Assessment of Chemical Composition

The most common analyses of PVRP performed and reported in various studies are blood cell counts, growth factor and chemokine concentrations and EV evaluation [89,90]. Blood cells can be counted manually with a hemocytometer [89] or determined with automated cell counting machines, usually based on flow cytometry [91,92,93,94]. The concentrations of growth factors and chemokines are usually determined by various immunoassays based on the principle of antigen-antibody reaction, e.g., enzyme-linked immunosorbent assays (ELISA) [46,92,93,94,95,96,97,98], bead-based cytometric immunoassays [98,99] and multiplex assays [45,92]. In addition, the presence of various proteins, such as growth factors, can also be assessed using the Western blot technique [100]. The concentration of various ions (e.g., calcium, magnesium, copper, zinc, iron, sodium, phosphate and potassium) and pH is measured using automated chemical analyzers that measure analytes using colorimetry, turbidimetry and electrochemical potentiometric ion-selective electrodes [92,101]. Gases in plasma are usually assessed with electrochemical sensors to perform potentiometric measurement of the pressure of CO_2_ and amperometric measurement of the pressure of O_2_ [101].

The elemental composition of PVRP can be measured by inductively coupled plasma optical emission spectrometry [102]. Various metabolites in PVRP samples can be determined by ion-parallel liquid chromatography (LC) and mass spectrometry (MS) or gas chromatography (GC) and MS [102], or by electrochemical sensors [103]. Hormones can be determined by liquid chromatography-electrospray ionization tandem mass spectrometry (LC-MS /MS) [103,104].

### 4.2. Assessment of the Quantity and Size of EVs

The quantity that reflects the size—the hydrodynamic radius R_h_—can be determined by dynamic light scattering (DLS) and interferometric light microscopy (ILM). In the isolate from plasma, the average R_h_ assessed by DLS and ILM was 39 nm and 68 nm, respectively [47], indicating the presence of a large proportion of lipoproteins and protein complexes in the samples. The number density of EVs in plasma as determined by ILM was about 10^11^/mL [47].

#### 4.2.1. Dynamic Light Scattering (DLS)

The focus on characterization of EVs is in the size range up to a few hundred na-nometers, where scattering of visible light, shortly light scattering (LS), has proven to be a very powerful technique for analyzing both size and also topology of particles [105,106,107,108,109,110,111,112]. For routine analysis, dynamic light scattering (DLS) is generally employed, leading to a value of the hydrodynamic radius (R_h_) of particles diffusing in a medium with a known viscosity. The advantage of DLS is that it is a reliable, non-destructive and rather simple technique, which is particularly suitable for the size-range of EVs. It is independent of particle type, and thus, allows analysis of complex fluids, such as blood plasma. On the other hand, the limitation of DLS is that it is a batch technique, meaning that the analysis is performed over all (a collection of) particles in solution. This is done by evaluating fluctuations of scattered light intensity (therefore, the term dynamic light scattering or DLS is used). The result of DLS is an average size (hydrodynamic radius, R_h_, or diameter, D_h_) of a population and its poly-dispersity. In the case of samples with more than one population that differ significantly in size, a distribution of LS intensity over all R_h_ values can be obtained for each population. Tarassova et al. [113] have introduced a method to deconvolute the total intensity of scattered light for each population based on size distributions measured by DLS and then analyze the scattering intensity of each population separately, a part of the static light scattering approach (see below).

An often-ignored point is that R_h_ in DLS is evaluated from the measured correlation functions via diffusion coefficient (D) by using the Stokes–Einstein equation [70,114], which requires knowledge of the viscosity of the medium. To obtain the correct value of R_h_, the true value of the medium viscosity is a crucial parameter.

In addition to DLS, static light scattering (SLS) enables the determination of another size parameter, the radius of gyration (R_g_) of particles, from measurements and analysis of the average LS intensity as a function of angle. SLS analysis is far from being a routine technique, which is probably the reason that it is not employed very frequently in EV research. However, by combining DLS and SLS results (R_h_ and R_g_, respectively), the shape of particles can be predicted by calculating so-called shape parameter ρ (= R_g_/R_h_). Parameter ρ has characteristic values, depending on the mass distribution within a particle, and therefore, on its shape/topology. For spherical particles such as vesicles of uniform size, in which the mass is concentrated on the rim (i.e., in the lipid double layer), the theoretical value of ρ is 1.

DLS in combination with SLS was recently used to analyze EVs isolated from blood plasma [113,114,115] using the deconvolution method of Tarassova et al. [113] and Sitar et al. [110]. In Sitar et al. [110] and Božič et al. [111], this approach was first tested by characterizing EVs in exosome standards, which were prepared as dilute suspensions of vesicles in water with a viscosity equal to 0.9 mPas at 25 °C. The same method was afterwards used also to analyze EVs in a much more viscous medium of blood plasma isolated from healthy donors, where viscosity proved to be a critical parameter in accurate R_h_ evaluation from the measured diffusion coefficient D (via the Stokes–Einstein equation). The viscosity of the medium where vesicles diffuse in blood plasma was for the first time estimated by Božič et al. [111] by direct measurements, and found to be around 1.2 mPa s at 25 °C, which is more than 30% higher than that of water. Simply using water viscosity in evaluation of R_h_ from D leads to a significantly overestimated value of R_h_, and therefore, an underestimated value of ρ. This point is often ignored in DLS measurements.

Two populations of EVs were identified in both exosomes standards and in blood plasma samples by DLS. For the subpopulation of small EVs (population 1 with R_h_ < 35 nm), which were classified as exosomes [110,113], no angular dependency of the LS intensity was obtained, which made it impossible to determine R_g_. This was attributed to the small size of these vesicles. It was also argued that the peak in R_h_ distribution for small EVs may be overlapping with the peak for proteins (R_h_ below 10 nm), thus leading to an underestimation of size. This presents a difficulty of batch techniques such as DLS and is sometimes overcome by using asymmetric flow field flow fractionation or AF4 [110,113], where separation of particles is achieved according to their size in a channel with a cross-flow prior to DLS analysis. Analyses of the subpopulation of larger vesicles (population 2) in an exosome standard [110,111] resulted in similar R_h_ and R_g_ values (in the range 135–170 nm), leading to excellent agreement of ρ (= 0.94–1.1) with its theoretical value of 1. Similar results with ρ in the range 0.94–1.1 were obtained for EVs in blood plasma of healthy donors [111] by considering the correct viscosity of water in R_h_ evaluation, as pointed above. A somewhat lower ρ was interpreted by considering the possibility that proteins or other smaller molecules are incorporated into the interior of EVs, thus decreasing the difference in density of the vesicle interior and shell.

Using SLS and DLS, sizes and structural characteristics were recently also studied for EVs found in plasma and ascites of patients diagnosed with advanced serous ovarian cancer (OC) [111]. In addition, this study enrolled patients with benign gynecological pathology (BP) as the control group. This presents the first detailed study of blood plasma of cancer patients. The majority of EVs in plasma and ascites of OC patients had R_h_ around 25 nm and ρ in the range of 0.97–1.16. Larger EVs (R_h_ close or above 100 nm) were found mainly in samples that underwent freezing and subsequent thawing, which are procedures required in the long-term storage of samples. The value of ρ was again similar (ρ = 0.99–1.15) to the smaller EVs, confirming the topology of hollow spherical particles. It was argued that the larger size of these EVs may be due to membrane rupture upon freezing and repeated organization of lipids into curved bilayer structures upon thawing, which points to the transient nature of assemblies such as vesicles. Mechanical or other stress (freezing/thawing) may lead to decomposition and repeated organization of lipids into bilayers, upon which larger vesicles may form. The data for BP patients were much more scattered, and differentiation between two EVs populations was not always possible. R_h_ and R_g_ values of EVs in BP patients were larger than in OC patients, with ρ mostly above 1, implying a more elongated/distorted shape in comparison with EVs of OC patients. Although not routine, SLS and DLS are promising methods for the analysis of morphological features of EVs and may have the potential to discriminate between OC and BP patients.

#### 4.2.2. Interferometric Light Microscopy (ILM)

In the recently developed method that is referred to as Interference Light Microscopy (ILM), the sample is illuminated by a LED light [47]. Interference enhances the information on the scattered light. The intensity of the interference signal is about three orders of magnitude higher than the intensity of the scattered light and the intensity of the incident light is about three orders of magnitude higher than the intensity of the interference signal. The source light interferes with the light scattered by a particle on the complementary metal–oxide–semiconductor chip located at the objective focal plane. To create the image, the contribution of the incidence light is subtracted from the detected image. The obtained pattern includes contrasting black and white spots that are recognized as particles which enable assessment of their positions. Number of particles within the volume considered is assessed. The interference pattern is processed to locate the particle and a video is recorded to track its movement. Particles with smaller masses in the same time move within a larger volume than particles with larger masses. The diffusion coefficient D of the motion of the particle is taken to be proportional to the mean square displacement d of the particle between two consecutive frames taken in the time interval ∆t, <d2(∆t)> = <4D ∆t>. The hydrodynamic radius R_h_ is determined by assuming that the particles are spherical by using the Stokes-Einstein equation. Each particle is tracked and processed individually. ILM has recently been installed into a commercially available instrument Videodrop (Myriade, Paris, France) and was hitherto used to analyze microorganisms in marine water [115], viruses [116] and extracellular vesicles [47,117]. ILM enables non-invasive assessment of number density and R_h_ of particles smaller than 500 nm, and therefore, assessment of diluted plasma samples directly without performing isolation that may considerably transform the constituents.

## 5. Storage of PVRP

To enhance the regeneration of tissues with wound healing impairment, PVRP would ideally be used in more than a single application. Despite recent optimized procedures of PVRP preparations, repetitive harvesting of concentrated platelets by blood withdrawal and centrifugation is time consuming and impractical for regular clinical practice. Potentially painful blood withdrawals and prolonged time of check-ups may result in lower compliance of the patient and worse treatment outcome. Therefore, in order to maximize the effect of PVRP in cases, where a repetitive application is beneficial, investigations of different storage conditions of platelet preparations have emerged. The focus of research is the preservation of preparations with retained growth factor, cytokine and chemokine activity. In PVRP science, platelet degranulation and release of functional proteins and their effect on vasculature, cell-growth and inflammation are the measured parameters of storage feasibility [118].

With the development of platelet preparations, the manner of storing platelet products to most optimally preserve the beneficial substances was concurrently explored. Until the 1970s, cold storage at 4 °C was the standard preservation technique for platelet concentrates, according to the arguments of decreased metabolic rate by which the product’s blood clotting abilities are preserved and bacterial growth inhibition is optimal [119]. It was later described that after transfusion, platelets at room temperature showed better in vivo survival [119,120,121,122]. In this regard, Murphy and Gardner (1969) first described the storage of platelets in plasma and proposed to change the convention of cold storage to preserving at 22 °C, for 4 days [122].

The stability of prepared platelet products at room temperature has been discussed in several studies. It has been reported that the preparations in the anticoagulated form are stable at room temperature for 8 h or longer after the preparation, enabling the use of preparations during lengthy procedures [123]. Marx et al. proposed that after the activation, the preparation should be used within 10 min, as nearly 100% of growth factors are released in the first hour. However, the platelets were found to still produce some bioactive molecules during the rest of their lifespan, which is 8 to 10 days [27]. Bausset et al., in their study on growth factors in autologous PVRP, recommended using the autologous PVRP concentrates at room temperature within 3 h after preparation, even though the concentrations of PDGF and VEGF were well-sustained up to 6 h at room temperature [123]. Wilson et al. reported that TGF-1 in PVRP retained its activity up to 4 h at room temperature [124]. The proliferative activity of room temperature-stored platelets was found to be retained for as long as 21 days, but the products are at risk for bacterial contamination or pyrogenic cytokine accumulation. Moreover, Moore et al. measured PDGF over a period of 8 days and concluded that the storage of PVRP for tissue regeneration at room temperature could be prolonged to at least 5 days [125]. Wen et al. similarly reported a sustained or even elevated level of growth factors after 7 days at room temperature storage of leukocyte-rich PVRP [126].

Due to the benefits of having platelet products at disposal for repetitive clinical use, a surge in interest for cold storage of platelet preparations has been noted in recent years. Several studies and their findings in investigating hypothermic platelet storage are presented in Table 3.

## 6. Use of Plasma Rich with Platelets and Extracellular Vesicles in Human Medicine

There is mounting research documenting autologous platelet products as a safe and effective therapeutic option used in a variety of medical fields and clinical settings. Outside of transfusion medicine, where platelet concentrates were used for blood loss and thrombocytopenia, the first field to apply PVRP was maxillofacial surgery, followed by many other fields, such as traumatology, orthopedic surgery, aesthetic surgery, dermatology, otorhinolaryngology, ophthalmology, gynecology, cardiovascular medicine, rheumatology and others.

### 6.1. Use of Plasma Rich with Platelets and Extracellular Vesicles in Treatment of Ligament and Tendon Injuries

Connective tissues (e.g., tendon, ligament and muscle), similar to other tissues, heal through the phases of inflammation, proliferation and remodeling [28]. The bioactive factors released from platelet products affect these metabolic processes through the established mechanisms of their effects on wound healing [30]. By restoring the vascularization of injured tendons, mobilizing circulation cells, influencing the proliferation of ligament cells and matrix synthesis, the products have the ability to enhance tissue regeneration. Many studies have investigated the effect of platelet preparations on tendons and ligaments, most commonly for chronic tendinopathies, acute ligamentous injuries, muscle injuries and intraoperative tissue augmentation [132]. In patients with lateral epicondylitis not responding to conservative treatment, PVRP has been used as an alternative to surgery, applied in a single injection. In 60% of patients, significant improvement, sustained over time, was observed with no reported complications [133]. Similarly, Sanchez et al. demonstrated enhanced healing of Achilles tendon tears by operative management combined with autologous platelet-rich fibrin matrices. The intervention group had earlier range of motion, no wound complications and patients were able to resume running sooner than the control group treated without platelet product application [134]. Thanasas et al. in a randomized control trial reported beneficial effects of platelet-rich plasma preparations for pain reduction in chronic lateral epicondylitis compared to other autologous whole blood products [135]. Gaweda et al. investigated the effect of autologous PRP injected into 14 non-insertional Achilles tendinopathy patients’ tendons, reporting a significant improvement in the clinical and imaging results at 6 weeks, and 3, 6 and 18 months after injection [136]. Moreover, Alviti et al. observed significant differences in biomechanical evaluation in favor of the interventional group of Achilles tendon surgical treatment with PVRP, versus the control group treated with operation only. Almost complete restoration of the biomechanics of gait was observed at 6 months after treatment in both groups, but the group of patients treated with PVRP augmentation resulted in additional significant functional improvements in ankle motion [137]. However, little to no effect was reported by other researchers in similar clinical applications. De Vos et al. reported no significant differences in treatment with PVRP in a double-bling randomized placebo-controlled trial for chronic Achilles tendinopathy [138]. In another randomized single-blind study by Schepull et al., autologous platelet preparations were found to have no effect on the healing of Achilles tendon ruptures [139]. Similar findings were recently described in a randomized, double-blinded prospective study by Boesen et al., concluding that the application of PVRP in non-surgically treated acute Achilles tendon ruptures did not show any superior clinical and functional improvement [140]. Filardo et al. systematically reviewed the most common tendinopathy conditions, concluding that PVRP injections most significantly positively affect patellar tendons, while the evidence for Achilles tendon, rotator cuff or lateral elbow tendinopathy did show improvement in most studies, but lack either statistically significant results or to demonstrate the advantage over other blood product applications [141]. Similar observations were reported in recent randomized control trial studies by Alsousou et al. and Keene et al. [142,143]. Hence, despite the promising results in separate case reports and case series, more randomized controlled trials are necessary to demonstrate significant benefit of treatment with platelet products in several orthopedic applications.

### 6.2. Use of Plasma Rich with Platelets and Extracellular Vesicles in Treatment of Chronic Wounds

Chronic wounds are by definition breaks in the skin that do not heal, require a long time to heal or frequently recur [144]. The most common chronic wounds are pressure ulcers, venous leg ulcers, arterial ulcers, neurotrophic ulcers and foot ulcers in people with diabetes [79]. Although chronic wounds are most commonly located on skin, they can also arise in mucosa. A chronic mucosal wound is for an example mandible osteoradionecrosis [145]. The most common risk factors contributing to poor wound healing are wound infections, repeated trauma, presence of necrotic tissue, systemic diseases (e.g., diabetes mellitus), tissue hypoxia (e.g., decreased arterial supply and/or venous outflow), immunodeficiency, neoplasms and medications (e.g., corticosteroids). The increasing number of chronic cardiovascular diseases and medication use worldwide, resulting in disrupted healing of tissues, have led to investigations of novel and supplementary techniques to standard wound care [144].

The first clinical demonstration of locally acting growth factors on chronic cutaneous ulcers was published by Knighton et al., naming the platelet preparation “Autolous Platelet-derived Wound Healing Factors” [1]. Stimulated healing rates were reported in patients treated with the preparation [1]. Early in the 1990s, Krupski et al. (1991) carried out a prospective randomized trial of chronic non-healing wounds with PVRP and showed significant stimulation in vascularized connective tissue [146]. Investigating diabetic ulcers, Margolis et al. published a retrospective analysis of 26,599 patients with diabetic neuropathic foot ulcers treated with an autologous platelet releasate. The results suggested that platelet releasate combined with standard care was more effective than standard care alone [147]. Similarly, Lundquist et al. demonstrated that PVRP incubated and activated with fluid from chronic diabetic foot wounds produced higher levels of growth factors than PVRP incubated with phosphate-buffered saline [148]. Investigating the same indication, Babaei et al. observed healthy granulation tissue formation and early complete closure of all wounds after PVRP topical application in 150 patients with diabetic foot ulcers [149]. Moreover, platelet preparations showed positive results in wounds secondary to AIDS. In a pilot study by Cieslik-Bielecka et al., leukocyte and platelet-rich plasma application enhanced neovascularization and reepithelization in patients with chronic crural ulcers with AIDS [150]. Since chronic wounds may persist as a result of tenacious infections, the antimicrobial activity of platelet preparations offers another aspect of its clinical benefit [24]. In a meta-analysis by Carter et al., therapy with platelet-rich products in cutaneous wounds demonstrated enhanced healing process and reduced infection rate compared to the control group, treated standardly [151]. In an analysis of chronic pressure wound surfaces, by Crovetti et al., a decrease in growth factor concentration was observed, compared to acute wounds [152]. Similar results from a different perspective have been reported by Yuan and colleagues, where the researchers demonstrated an increase in multiple growth factors in the granulation tissue of refractory diabetic ulcers after PVRP treatment [153]. McAleer et al. reported full wound closure and epithelization in 20 of 33 chronic lower extremity wounds after failed conservative and surgical treatment. The wounds were injected with PVRP every 2 weeks [154]. Martinez-Zapata et al. analyzed randomized controlled trials (RCTs) that compared autologous PVRP with a placebo or with other treatments for any type of chronic wound in adults. They stated in the analysis that despite the low-quality evidence from two small RCTs, PVRP may improve the healing of foot ulcers associated with diabetes, but found no strong evidence of the positive effect of PVRP in other types of chronic wounds, as the majority of studies inefficiently detected the treatment effects or exerted high risk of bias [79].

### 6.3. Use of Plasma Rich with Platelets and Extracellular Vesicles in Treatment of Burns

A burn wound differs from non-burn wounds in the fact that it is edematous, al-ready contains plenty of activated platelets with boosted inflammatory response and has restricted blood flow to the wound due to high coagulability in the burn. A specific time-dependent concentration of platelets has been shown in patients with wound(s). This emerged in ideas of alteration of the composition of PVRP and/or timing of its application in burn patients [155,156].

There are several studies discussing platelet products and burn wounds. In a study by Kazakos et al., PVRP was applied on acute wounds, 19% of which were friction burns. Statistically significantly accelerated healing was observed in burned tissues after the application of the preparations [157]. Zheng et al. published a systematic review on the effectiveness of PVRP in burn wound healing, which included 13 studies: three studies were randomized-controlled trials and the rest were prospective or retrospective studies. The review concluded that PVRP exerts a treatment potential in reducing blood loss, prolonging the viability of skin grafts, accelerating the rate of healing in minor burns and improving scar tissue quality after wound healing. According to the reviewed literature, they found no significant differences in graft take, degree of epithelization, pain severity, adverse reactions and infections between the PVRP and control groups [155,158]. However, a possible disadvantage of the preparation was noted by some authors. As one of the effects of platelet products is to increase fibroblast proliferation, a concern of hypertrophic scar formation was raised. In the study, no enhanced reepithelization was found, but a significant increase of vascularization and fibroblastic proliferation was evident [156,159].

### 6.4. Regenerative Effects of Plasma Rich with Platelets and Extracellular Vesicles in Maxillofacial Surgery, Dental Medicine and Bone and Joint Disorders

With its pool of growth factors, cytokines and other molecules essential for bone growth and proliferation by mimicking bone healing conditions, platelet products present a favorable treatment option in regenerative medicine [160,161]. Aiding the regeneration of bone tissue is especially important in diseases involving bone loss, such as periodontitis, tumors, fractures and bony defects. Many in vitro studies have shown a significant relationship between the application of PVRP and the proliferation of adult mesenchymal stem cells, the proliferation of fibroblasts and the production of extracellular matrix [71]. Amid most investigated fields of PVRP use are the regeneration and reconstruction of skeletal and connective tissues in the periodontal and maxillofacial pathologies [162]. As a group of pioneers in the field, Marx et al. reported the results of a randomized control trial with 88 patients receiving cancellous marrow bone graft with or without PVRP, documenting the positive effects of the preparations [160]. Since then, it has become common in oral and cranial surgery to combine platelet-rich material with autograft, allograft, demineralized bone matrix or other graft material to fill bony defects in the mandible or other bones in the cranium [163]. Other applications in maxillofacial regeneration have been described. Cieslik-Bielcka et al. demonstrated a faster callus formation and oral mucosa healing after the removal of odontogenous cyst of the mandible after platelet gel application [150]. Additionally, beneficial effects of PVRP have been described in mandibular fractures, bisphosphonate mandibular osteonecrosis combined with necrectomy, periodontal tissues healing in periodontal disease and in the stability of dental implants [150,163,164].

Additionally, the application of PVRP for regeneration purposes has given promising results in acute and chronic injuries of bone and cartilage [165]. The benefits of autologous platelet products compared to other options (e.g., autogenous bone grafting) are its availability, ease of isolation, storage properties and eliminated risk of disease transmission and immune reaction in autologous preparations [161,165]. In papers researching bone regeneration, Kon et al. reported the results of 91 patients (115 knees) treated with PVRP, which was found as a safe therapeutic option, superior to hyaluronic acid supplementation. The reduction of pain and knee function improvement was seen, especially in younger patients; however, the effects were only temporarily observed [166]. In another study by Lowery et al., PVRP and autogenous bone grafts were administered during lumbar spinal fusion resulting in complete union in all their patients [167]. Bielecki et al. similarly investigated the use of percutaneous injections of autologous platelet-rich gel as treatment of delayed and nonunion bones of 32 patients. The results revealed 12 had delayed union and 20 nonunion, with union achieved in all cases of delayed union after platelet injections [168]. Recently, Belk et al. conducted a systematic review and meta-analysis of randomized control trials for PVRP versus hyaluronic acid for knee osteoarthritis, demonstrating a significantly higher mean improvement score in patients treated with platelet products. The researchers emphasized the superiority of leukocyte-poor PVRP for this treatment [169]. Berney et al. analyzed data on PVRP injections for hip osteoarthritis, concluding better patient outcome scores at follow-up at 6 and 12 months, but no significant difference between patients treated with PVRP or hyaluronic acid alone. They suggested further studies should be conducted comparing intraarticular PVRP and steroid injections, the only intraarticular injection recommended by international guidelines for the treatment of hip osteoarthritis [170].

### 6.5. Use of Plasma Rich with Platelets and Extracellular Vesicles in Treatment of Ocular Surface Disorders

The use of blood-derived eye drops, especially serum eye drops (SED), has become increasingly popular in recent decades for the treatment of ocular surface disorders. In the normal eye, the tear film plays a crucial role in maintaining the health of the ocular surface epithelium, providing antibacterial, epitheliotrophic factors and nutrients for the cells [171]. In the serum, several growth factors, fibronectin and vitamin A concentrations were detected at higher concentrations than in natural tears, suggesting a possible therapeutic option for persistent epithelial deficits [172,173]. Platelet products have been proposed to aid by the mechanism of stimulation of cellular proliferation and migration by supplying an active mixture of growth factors and cytokines at the ocular surface, mimicking the function of the lacking natural tears and aiding the healing process [174,175].

In the past decades, several in vitro and in vivo studies have shown platelet products to be favorable for ophthalmologic disorders [176,177]. Blood-derived eye drops have been used for the treatment of dry eye disease, persistent corneal epithelial defect, corneal ulcer, chemical burn, recurrent corneal erosion and limbal stem-cell deficiency [174]. Persistent epithelial defects (PED) of various etiologies (i.e., post-refractive surgery, post-penetrating keratoplasty), recurrent corneal erosions and neurotrophic keratitis have also been explored [178,179,180,181,182]. One of the first studies utilizing PVRP on ocular surface disorders was by Ralph et al. in patients with chemical burns [183]. The research was followed by the use of autologous serum eye drops in patients with Sjögren’s syndrome-related dry eye [184]. In a review by Giannaccare et al., five prospective randomized controlled trials were analyzed, all of which reported improvement of symptoms and tear film break-up time, as well as other parameters [174], while in one of the studies [185], no significant differences between the intervention and the control groups were found. The analyzed randomized control trials reported promising results on autologous SED treatment, although, previously, a Cochrane Database review by Pan et al. failed to show long-term efficacy of the preparations in dry eye disease [174,185,186]. Lekhanont et al. conducted a large prospective study with 181 patients receiving SED for persistent epithelial defects secondary to ocular surgery and reported complete corneal epithelialization within an average of 4 days in more than 90% of patients [180]. SED has also been shown encouraging results in treatment of diabetic patients with corneal deficits [179]. Alio et al. additionally, demonstrated positive effects of the preparations in managing the dry eye syndrome symptoms. In total, 89% of their examined subjects reported a significant improvement in dry eye symptoms after PVRP treatment [187]. Kim et al. applied PVRP on persistent corneal epithelial defects, reporting an accelerated healing rate compared to the control group [178]. Alio et al. (2007) treated 40 patients with dormant corneal ulcers with PVRP and demonstrated diminished pain severity with stable or improved vision in all patients [188]. Alio et al. (2013) treated 11 cases with persistent corneal perforation, adding both fibrin membrane and PVRP prior to corneal grafting. The results revealed sealed perforations and concluded that PVRP therapy is a safe and effective surgical alternative for the closure of corneal perforation as long as the corneal tissue permits definite surgical intervention [189]. Moreover, it was suggested that PVRP can be used not only as a topical eye drop, but also as biomaterial used for reconstruction procedures [189].

Another point of interest of platelet product use in ophtalmology is the corneal injury management. Panda et al. showed significant improvement in visual acuity and corneal transparency after 3 months of therapy with PVRP eyedrops along with standard medical care [190]. More recently, the research has concentrated on the exploration of effective storage of eye drops.

Anitua et al. demonstrated in their study that PVRP eye drops can be stored for up to 3 months without any reduction of the main proteins involved in ocular surface healing [171]. Lopez-Garcia et al. measured growth factor concentration in autologous eye drops, which remained stable over the 4 weeks at 4 °C, both in fresh and in defrosted samples; no statistically significant differences were found between the growth factor concentration in fresh samples and samples after 1, 3, 6 and 9 months of freezing at −20 °C. The storage did not produce differences in the cell proliferation and differentiation between cultured cells treated with fresh samples or samples saved 4 weeks at 4 °C or defrosted after 1, 3, 6 or 9 months at −20 °C [127]. It was found that platelet lysates can be stored at −15 °C for up to a month, retaining their beneficial properties [191].

### 6.6. Use of Plasma Rich with Platelets and Extracellular Vesicles in Scar Revision

The potential of platelet products to improve the condition of scar tissues after cutaneous injuries has been explored in several studies [192]. The efficacy of preparations is most commonly measured by scar severity scales, patient-orientated questionnaires and the volume of tissue gained [73]. In the setting of traumatic scars, Azzena et al. described the use of PVRP as a delivery system for adipose implantation, for the platelet molecules to stimulate recruitment of microcapillaries at the implantation site. Autologous fat was included into the platelet preparation and injected into a painful shoulder scar in a patient. Using histology, immunohistochemical analysis and ultrasound, beneficial results were observed. The researchers described prolonged survival of a fat pocket 1 year after surgery [193]. Cervelli et al. studied the effect of fat grafts mixed with leukocyte rich-PVRP on traumatic scars in combination with non-ablative laser skin resurfacing. The group of patients who received leukocyte rich-PVRP and fat tissue resulted in improvement in two out of four points on the specific scale to assess scar tissue. The most effective, three-point improvement was observed in the combination of leukocyte rich-PVRP, fat and laser group. The separate effect of PVRP was not assessed, which was a disadvantage of the study [85]. Majani et al. examined the benefits of PVRP in scar tissue using lipografting with PVRP and lipografting without PVRP. They assessed no quantitative scar outcomes to support the hypothesis of platelet products aiding scar tissue regeneration [194]. Gentile et al. investigated the use of adipose-derived stromal vascular cells and PVRP in patients with scars on the face, due to burns and trauma. The control group were patients treated with centrifuged fat only. The first group displayed a significantly higher maintenance of contour restoring after 1 year compared to the control group [195].

Platelet products have also been evaluated for their potential benefits in the treatment of acne scars. In a non-split face study by Zhu et al., topical platelet gel was applied to the facial skin treated with erbium fractional laser for acne scars. In total, 91% of patients demonstrated a 50% or more improvement on their scars after repetitive treatment [196]. Lee et al. conducted a split-face study, injecting leukocyte rich-PVRP injections on one side of the face after carbon dioxide laser therapy for acne scars. They reported a reduced overall duration of erythema and an improved clinical appearance of acne scars in the PVRP group [197]. Comparison of leukocyte rich-PVRP injections and topical application in laser treatment for acne scars was investigated by Gawdat et al. in a two-group split-face study [198]. Shorter recovery times and significant improvements in clinical appearance of scars were found in the investigation group compared to the control group, with intradermally injected and with topically applied PVRP. No significant differences between the topical and intradermal platelet product application were observed, but the patients tolerated topical preparations better [198]. Na et al. examined the effect of PVRP after ablative fractional carbon dioxide laser resurfacing. A significantly faster recovery of the skin on the PVRP-treated side. They also observed a lower erythema index, melanin index and thicker collagen bundles in the biopsy specimens from the PVRP-treated compared to the control side [199]. Asif et al. compared PVRP and micro-needling for atrophic scars compared to micro-needling only and concluded that PVRP enhanced the micro-needling effect [200].

Gupta et al. reviewed eight studies examining the effect of PVRP on scar tissue and found that only four of them were high quality, placebo-controlled, randomized and blinded trials [73]. In these studies, PVRP was shown to statistically significantly improve acne scars when combined with micro-needling and with erbium fractional lasers, but did not outperform results of trichloroacetic acid combinations or CO_2_ fractional laser treatments [201,202,203].

### 6.7. Use of Plasma Rich with Platelets and Extracellular Vesicles in Treatment of Alopecia

It was suggested that the most effective method of application of PVRP for hair restoration were subdermal injections, as they allowed the delivery of bioactive molecules directly to the base of the hair follicle and spreading into the subdermal space or interstitium [204]. One of the earliest articles on PVRP for androgenic alopecia (AGA) was published by Uebel et al., who reported a 15% increase in follicular unit density in areas treated with PVRP compared to the control areas of the scalp [205]. In an analysis of AGA by Gupta et al., it was concluded that monthly PVRP treatments (first three sessions, followed by a maintenance regimen) can significantly improve hair density, hair count, hair shedding and hair diameter in patients with a mild condition while in patients with severe hair the treatment failed to produce a beneficial effect [73]. The use of three sessions was recommended by Picard et al., who reported a progressive effect of PVRP from the first injection. The effect reached a peak after three to five injections [206]. In a recent meta-analysis by Atiyeh et al., it was concluded that in addition to total hair density, PVRP induced improvement in hair count, terminal hair density and hair shedding. They confirmed that PVRP could also be a beneficial adjunct to hair transplantation [207]. Similarly, in a large systematic review by Chen et al., 21 of 24 studies examining the effect of PVRP on hair restoration reported positive outcomes (88%), both subjective and objective [208]. However, in another meta-analysis by Gupta et al., comparing PVRP with other approved nonsurgical AGA treatments, low-level laser therapy was considered the superior treatment to other therapeutic options, including platelet products [209].

### 6.8. Skin Rejuvenating Effects of Plasma Rich with Platelets and Extracellular Vesicles

There is a significant interest of researchers on the effect of PVRP on the aging skin. Platelet product applications have been widely investigated in aesthetic medicine for their beneficial use in facial skin procedures and skin aging by improving collagen fiber density via activating fibroblasts [210]. The products are applied onto the skin topically or injected intradermally. Contrary to what has been shown for hair restoration, no significant differences in effect on the skin were observed between topical and injected applications [73,204].

In a review by Gupta et al. assessing the quality of platelet-product application studies, 57% of aging facial skin studies and 67% of facial procedure studies were graded as low quality or moderate quality. It was concluded that the most positive effects of PVRP are based on clinical case reports and case series of inconsistent quality [73]. The studies of Hersant et al. found no effect of PVRP in facial aesthetics [211].

The most statistically significant effect of PVRP reported by several authors is the improved appearance of nasolabial folds, photoaging and infraorbital circles [211,212,213]. Additionally, PVRP proved useful in lipofilling procedures. Elnehrawy et al. and Sclafani et al. demonstrated a significant difference in nasolabial fold appearance improvement after PVRP applications [212,214]. Mehryan et al. explored the effect of PVRP intradermal injections on crow’s feet wrinkles and infraorbital dark circles and found a significant improvement in infraorbital color homogeneity, while no significant changes were observed in the wrinkle volume and visibility index, among other parameters [215]. Interestingly, in the area of infraorbital rejuvenation, Neinaa et al. observed that platelet-poor gel injections were more effective than PVRP [216]. In a randomized split-face clinical trial, Alam et al. studied the effect of PVRP injections but observed no difference between the PVRP and saline-infused halves of the face for fine wrinkles. However, they observed a statistically significant difference in the effect of PVRP on photoaging [217].

Several studies investigated a synergistic effect of PVRP and other procedures or agents. According to a study by Willemsen et al., it has been observed that PVRP significantly decreased the number of recovery days and improved the facial volume and appearance combined with lipofilling procedures, compared to lipofilling alone [218]. Beneficial combination of topical PVRP application on the skin previously treated for resurfacing with electroporation or CO_2_ laser has been observed in a study on perioral wrinkles [219]. Shin et al. demonstrated a complementary effect of PVRP and fractional laser treatment for skin rejuvenation. An increase in collagen density and fibroblast count, lower erythema index and objective improvement of skin elasticity were observed [220]. Abuaf et al. performed histopathological analysis of skin biopsies in the investigation group treated with PVRP and the control group treated with saline injections. They demonstrated greater increase in dermal collagen levels in the PVRP-treated group; the collagen level also increased in the saline-treated group, suggesting that dermal thickness is increased not only due to PVRP but also due to skin needling [210]. While nearly all published reviewed literature found some anti-aging potential of PVRP, despite not being able to find statistically significant differences between the investigation and control groups, a study by Yuksel et al. reported fibrosis rather than skin regeneration in the dermis after PVRP injection. The authors emphasized that the observed inflammation and microangiopathy could lead to trophic alteration of the skin, which does not contribute to the anti-aging goal [221].

The aesthetic rejuvenation potential of PVRP especially in combination with other procedures and modalities, remains hypothetical. More randomized comparative studies and clinical trials are needed for definite conclusions on facial rejuvenation.

### 6.9. Use of Plasma Rich with Platelets and Extracellular Vesicles in Otorhinolaryngology

Platelet preparations have been explored as promising material in managing several otorhinolaryngological conditions. Their use has been reported in nearly all anatomical units of this field. In the laryngeal surgery, the underlying idea is to reduce excess collagen deposition in vocal fold scar tissue, interact in angiogenesis pathways and aid in tissue regeneration [222]. A review of laryngeal applications of PVRP was published by Suresh et al., where platelet products (PVRP or platelet-poor plasma) were injected in patients with idiopathic unilateral vocal fold palsy, glottic insufficiency after chest or thyroid surgery, uni- or bilateral vocal cord scars, vocal cord sulcus and vocal cord atrophy [222,223,224]. The summary of the studies revealed beneficial healing properties of PVRP and platelet-poor plasma on human and animal laryngeal tissue by upregulating growth factors and inducing neovascularization, which was clinically shown as improvement of objective parameters of the voice, subjective patient satisfaction and medialization of vocal folds, especially in acute laryngeal injury [225]. The injections proved to be safe, with no foreign body reactions seen on injection sites. As adverse side effects, injection-site hematoma in an anticoagulated patient [226] and 1–3 days of postoperative throat pain [222] were observed.

Platelet products have also been the subject of interest in otology, especially in tympanic membrane healing disorders. The suggested benefit of PVRP, applied in animal and human, is in accelerating the healing and preventing dehydration of perforation margins [226,227]. A review of published articles on the topic has been done by Huang et al., who reported 93.4% of complete closure cases in patients that received PVRP after the surgery compared to 78.6% complete closure cases in patients that had surgery alone, with low incidence of complications [228]. In one case, graft rejection was reported in a patient with surgical repair failure. In the same study, PVRP bactericidal properties were suggested, since four cases in the control group had postoperative infections and none of those with PVRP application [229]. Navarrete Alvaro et al. reported adjuvant use of PVRP in tympanoplasty with complete closure of tympanic membrane perforations [230]. Sankaranarayanan et al. found that the PVRP clot application during tympanoplasty prevented graft displacement [231]. PVRP preparations were shown to facilitate healing in acute tympanic membrane perforations, as shown in a 32-patient study [232]. Elbary et al. demonstrated positive results using titanium mesh and PVRP mixed with bone material to reconstruct the posterior meatal wall after canal wall-down mastoidectomy for middle ear cholesteatoma [233]. PVRP along with continuous hyperbaric oxygen and polydeoxyribonucleotide was used in a unilateral complete ear amputation. It has been demonstrated that PVRP successfully salvaged almost the entire auricule [234]. It was confirmed in a randomized controlled clinical trial by Vozel et al. that autologous PVRP is an effective treatment modality for chronic postoperative temporal bone cavity inflammation in patients whose disease could not be treated surgically to maintain serviceable hearing loss and a reasonable disease-related quality of life [74].

Several publications about platelet preparations used in rhinology and in skull-base surgery reported the effectiveness of the treatment. Friji et al. described encouraging subjective and objective results in five patients with atrophic rhinitis, treated with PVRP and a fat transplantation method applied to the nasal mucosa with injections. Less nasal crusting, glistening of the mucosa and atrophy signs were observed [235]. Kim et al. used PVRP injections in 22 patients with atrophic rhinitis and reported significantly decreased NOSE (Nose Obstruction Symptom Evaluation) and SNOT-22 (Sino-nasal Outcome Test) scores, which corresponds to the improved disease-related quality of life [236]. Moreover, platelet preparations have been used as complementary materials in surgical treatment of sino-nasal diseases. Pomerantz and Dutton compared patients that underwent endoscopic sinus surgery complemented by packing with platelet gel to patients in which traditional packing was applied. Platelet gel offered more comfort and enhanced hemostasis [237]. Salaheldin and Hussein considered a group of 30 patients treated with PVRP by injection into the inferior turbinates after submucosal diathermy. They found significant improvements in crusting and bleeding, as well as mucocilliary clearance [238]. Kuzucu et al. conducted a study of 25 patients receiving standard nasal packs infused with PVRP and reported lower NOSE scores, less bleeding and crust formation compared to the control group, where the subjects received only saline nasal packing, confirming the hypothesis of accelerated healing in the septal and conchal mucosa [239].

The use of PVRP In anterior skull base surgery has been reported, most often presenting the author’ experience with cerebrospinal fluid leaks. Soldatova et al. administered leukocyte and platelet rich fibrin (L-PRF) on 47 patients additionally to sellar, parasellar and suprasellar benign and malignant disease [240]. Similar preparation was used by Khafagy et al., who applied L-PRF onto the tissues during transnasal closure of CSF leak [241]. In a pilot study by Fredes et al. in patients with osteonecrosis of the skull base, in four of five cases, new bone formation was evident on imaging [242]. Rasmussen et al. similarly used L-PRF and reported lower rates of crust formation [243]. Similarly, in a recent RCT study by Constanzo et al., 103 patients who underwent an endoscopic endonasal approach and were reconstructed using a standard technique were compared with 139 patients using the same protocol with the addition of L-PRF, postoperative intracranial infection and CSF leak at 6 months. The researchers found that PRF reduced the rate of postoperative CSF leaks in patients with intraoperative leaks, without differences on postoperative meningitis [244].

Olfactory neuro-epitelium and olfactory fillae have the ability to regenerate; thus, injections and packing of platelet products may promote axon regeneration by upregulating growth factors, acting anti-inflammatory and regeneratively. PVRP has been administered to the olfactory cleft [243]. Mavrogeni et al. applied four PVRP injections in 4-week intervals to five patients, who all improved in olfactory sensations after treatment with PVRP [245]. Especially in a COVID-19 and post-COVID-19 era, the interest of olfactory disfunction physiology and treatment surged. The anti-inflammatory and regenerative potential of PVRP offered an option of treat persistent viral inflammation of neuroepithelium. Injections of PVRP into the olfactory area for COVID-19 patients with persistent olfactory dysfunction were administered by Lechien et al. and Steffens et al. [246,247]. Both studies showed encouraging results, with most patients reacquiring the sensation of smell.

PVRP was tested in the pediatric otorhinolaryngology cases. Sidman et al. applied PVRP to the tonsillar fossae after tonsillectomy on 35 children and demonstrated no significant difference in pain, recovery time between the treated group and the control group receiving no specific topical treatment post-operatively [248]. Nanditha et al. reported PVRP as an effective treatment in accentuating healing and reducing post-tonsillectomy pain and morbidity [249].

## 7. Use of Plasma Rich with Platelets and Extracellular Vesicles in Veterinary Medicine

### 7.1. Use of Plasma Rich with Platelets and Extracellular Vesicles in Treatment of Ligament and Tendon Injuries

There are contradictory statements in the scientific literature about the effectiveness of the use of PVRP in the treatment of ligament and tendon injuries in veterinary medicine. Some researchers claimed that PVRP was effective for treatment of ligament and tendon injuries [250,251,252,253], while others found no evidence about the effectiveness of PVRP [254,255].

The results of Waselau et al. suggest that horses with moderate to severe midbody suspensory ligament desmitis treated by means of a single dose intralesional injection of PVRP, followed by a program of gradually increasing exercise, had an excellent prognosis for returning to racing [250]. PVRP can be expected to yield favorable therapeutic responses in Western performance horses with lameness localized to the proximal suspensory region [251]. In the study of Geburek et al., a single intralesional treatment with PVRP up to 8 weeks after onset of clinical signs of tendinopathy contributed to an earlier reduction of lameness compared to saline treatment and to an advanced organization of the repair tissue [252]. It was found that the fibrillar matrix organized into fascicles during the remodeling. Long term PVRP treatment was suggested to have the potential to increase the number of horses reaching their previous level of performance and early treatment of tendinopathy with PVRP should be considered [252]. The study of Scala et al. concluded that treatment with platelet-derived growth factors led to the formation of a tendon with normal morphology and functionality [253].

A systematic review and meta-analysis of clinical and experimental data about the use of PVRP for treatment of tenodesmic lesions in horses found no evidence that PVRP enhances the healing of tendon and ligament injuries [254]. In the future, further unbiased, blinded and controlled studies are needed to clarify the efficacy of this platelet concentrate in the treatment of equine tendon and ligament injuries [254].

A comparative systematic review on clinical and experimental data on PVRP treatment of equine and human musculoskeletal lesions [255] indicated that PVRP has the potential to exert beneficial effects in the healing of tendons, ligaments and cartilage, but definitive clinical evidence of its efficacy remains lacking. The results of Brossi et al. confirmed that biased, poorly designed studies, which are not properly controlled or blinded or adopt inadequate outcome measures, favored the observation of positive results; it was suggested that the majority of equine clinical studies that tended toward positive outcomes lacked randomization, blinding, adequate statistical power, outcome measures and control groups yielded positive results [255].

### 7.2. Use of Plasma Rich with Platelets and Extracellular Vesicles in Treatment of Arthritis

Osteoarthritis is one of the main causes of musculoskeletal disabilities in horses, and currently, there is no effective treatment for this disease. Treatments of osteoarthritis that focus on the modulation of inflammation and disease progression offer a new hope. For this reason, PVRP-derived products have been used to enhance healing of musculoskeletal injuries and modulate progression of inflammatory processes, including osteoarthritis. Several factors related to PVRP production, including methods of preparation and application, and intraindividual variability, lead to an inconsistent product, precluding reliable conclusions about its efficacy for clinical use [256].

The results of the study of Mirza et al. indicated changes in kinetic gait parameters following intraarticular administration of autologous PVRP in horses with naturally occurring forelimb osteoarthritis. It was suggested that there may be a potential benefit of autologous, intraarticular PVRP therapy for forelimb osteoarthritis in some horses [257].

The report of Tyrnenopoulou et al. found that intraarticularly injected autologous platelet lysate was efficient for temporarily managing osteoarthritis of the distal interphalangeal joint in athletic horses. Horses in this study remained sound for a period of at least six months while being in training. One year post-injections, however, all horses relapsed to their initial degree of lameness [258].

### 7.3. Use of Plasma Rich with Platelets and Extracellular Vesicles in Wound Healing

Carter et al. treated wounds with PVRP gel. It was found that PVRP gel induced accelerated epithelial differentiation and produced tissue with organized, interlocking collagen bundles [259]. It was suggested that the novel natural wound-healing agent induced wound repair in injuries that were previously deemed untreatable [259]. It was suggested that topical treatment with autologous PVRP gel, as additional therapy, might be considered beneficial in the management of large wound healing in horses and that it can be regarded as a safe and inexpensive treatment that can be used in field [259].

It was reported by Pereira et al. that the treatment of distal limb skin wounds with PVRP gel reduced the healing time of the wounds compared to the treatment of wounds in the control group with standard treatment; PVRP gel showed the best result on wound healing of the distal limb of horses in both clinical and histopathological evaluations [260].

It was found that PVRP induced excessive development of granulation tissue (assessed 1, 2 and 3 weeks after the appearance of the wounds on the distal aspect of the forelimb in horses) and significantly slowed down wound healing [261]. Topical application of autologous PVRP may be better suited to treat wounds with massive tissue loss, or alternatively, chronic wounds that would benefit from a fresh source of mediators to accelerate the healing process [261].

### 7.4. Use of Plasma Rich with Platelets and Extracellular Vesicles in Treatment of Burns

It was suggested that treatment with two applications of PVRP gel accelerated the repair of the extracellular matrix and its components in deep burn wounds in horses, but had a potential of fibroses formation [262]. PVRP gel exhibited also antibacterial activity in burn wounds and prevented further complications due to contamination [262].

### 7.5. Use of Plasma Rich with Platelets and Extracellular Vesicles in Treatment of Corneal Disorders

Beneficial effects of PVRP on proliferation as well as migration capacity of equine corneal cells were observed in vitro [263].

## 8. Use of Plasma Rich with Platelets and Extracellular Vesicles and Stem Cells for Regeneration

Research results from the last two decades showed that mesenchymal stem cells (MSCs) are found in all organs of adult organism and are responsible for organ repopulation and cell-turnover, enabling its vitality and integrity. For the purpose of tissue regeneration the most popular sources of MSCs are adipose tissue, bone marrow, umbilical cord, synovium, dermis, periodontal ligament and dental pulp [264].

There are several approaches when stem cells are considered as therapeutic: (1) to use pure, homogenous population of stem cells; (2) to use of various scaffolds that enable concentrating and positioning of cells; (3) or to use mixed population cells, including progenitor cells and growth factors. Obtaining stem cells in high number and purity is usually time-consuming and expensive procedure that also needs high grade sterility and complex infrastructure. However, it ensures a well-characterized and controlled product, which could be defined through criteria proposed by The International Society for Cellular Therapy [265]. Nevertheless, cells obtained in this procedure cannot meet criteria of “minimally manipulated product”, which is given by regulatory frameworks among Western countries, thus disabling wide usage of this approach. For that reason, usage of PVRP as a vehicle for delivery or during cultivation of stem cells as a source of growth factors has become a widely used approach. When PVRP is added to MSCs, it helps as a vehicle to confine cells in the chosen site, and also to enhance biological actions of implanted cells [266]. This combination enables the usage of lower number of stem cells for therapeutic effect, enabling the usage of non-passaged cells, such as stromal vascular fraction. Although their preparation, mechanism and action and efficacy have been shown to be different, studies have shown that both PVRP and stem cells can complement each other and might have an added advantage when used in combination [161,266,267]. Combined repair of the tissue occurs through trophic support, enhanced anti-inflammatory and immunomodulatory functions and increased differentiation capacity [265,267]. PVRP offers a suitable microenvironment for MSCs to promote proliferation and differentiation and accelerates healing capabilities.

Combined application of PVRP and stem cells showed good therapeutic effects in bone healing [161,268], osteoporosis [266], ovarian rejuvenation [269], skin wound healing [270] and more.

Besides combining PVRP and MSCs into a single treatment, PVRP can be used during laboratory cell expansion as a substitute of fetal bovine serum or autologous serum [270,271,272,273,274]. It is important to emphasize that PVRPs obtained through different methods showed differences with regard to their differential potential [274]. In addition, the number of platelets in the PVRP can have repercussions on the fate of MSCs [275].

## 9. Conclusions

According to the literature findings reviewed herein, PVRP represents a promising therapeutic option for several pathophysiologic states in human and veterinary medicine. It also gives an alternative option in reproductive medicine for ovaries rejuvenation, in emergency medicine for treatment of burns and bone fractures, as well as in otorhinolaryngology, head and neck surgery, dental and aesthetic medicine. Several advantages, such as low cost and relatively fast preparation, makes this procedure very practical for usage ex tempore.

Considered a safe procedure, since it is autologous, it carries a low risk of complications, such as infections or immune rejection. However, it is not yet clear which components are key in the healing process and what are the underlaying mechanisms. Additionally, as blood is a dynamic material, the effects of preparation procedures are yet poorly understood. Better understanding of these effects is expected to lead to optimized protocols for PVRP preparation. For that, further investigation is necessary. We believe that future explorations should be focused on developing patient-individualized protocols, as a single protocol cannot fit all patients. Additionally, controlled clinical studies with sufficient number of patients should be undertaken in to allow for the assessment of statistical and clinical relevance of the treatment with PVRP.

## Figures and Tables

**Table 1:** Granule contents of platelet molecules and their suggested involvement in healing mechanisms.

Biocactive Molecule	Suggested Healing Mechanism
Molecules originating from α-granules	
PF4	Chemokine: monocyte, recruitment of neutrophils and lymphocytes, differentiation of helper T-cells
PPBP	Chemokine: recruitment and activation of neutrophils; activation of macrophages
CCL5 or RANTES	Chemokine: recruitment of immune cells
P-selectin	Adhesion transmembrane protein: mediation of the adhesion of leukocytes; activation of complement
CD40L	A TNF receptor superfamily: provision of activation signals in antigen-presenting cells such as B cells, macrophages and dendritic cells
TGF-β	Cytokine: promotion of T lymphocyte proliferation; involvement in regulation of B lymphocytes and macrophage proliferation
PDGF	Growth factor: influence on growth and differentiation of monocytes and macrophages; enhancement of cell migration and proliferation; improving cell survival associated with crosstalk with extracellular matrix components and receptors
vWF	Glycoprotein: mediation of the adhesion of platelets; extravasation of neutrophils
FGF	Growth factor: promotion of growth, differentiation and motility of cells; mediation of angiogenesis by interaction with heparin, heparan sulfat and proteoglicans to induce action of heparan sulfate-degrading enzymes
VEGF	Growth factor in the family of pdgfs: induction of angiogenesis; permeabilization of vessels; recruitment of inflammatory cells; expression of adhesion molecules
IGF-1	Growth factor: promotion of migration of stromal cells into the fibrin clot; stimulation of proliferation of fibroblasts and endothelial cells; modulation of cell apoptosis
Thrombospondin	Matricellular glycoprotein: regulation of cell migration, cellular attachment and invasion
MIP-1α	Cytokine: activation of neutrophils and eosinophils; formation of immunoglobulins
MMP-2, MMP-9	Proteases: degradation of extracellular matrix; formation of platelet and leukocyte clusters
cyclophiline A	Smooth muscle growth factor
Molecules originating from δ granules	
Serotonine	Biogenic amine: regulation of dendrite cells and lymphocytes T, vasoconstriction and increase of capillary permeability
Glutamate	Amino acid, neurotransmitter: regulation of lymphocytes T
ADP	Adenine nucleotide: activation of platelets, leukocytes and endothelial cells
Histamine	Biogenic amine: degranulation, increasing vascular permeability, involvement in pro- and anti-inflammatory effects
Molecules not originating from granules	
IL-1β	Cytokine: involvement in acute inflammation phase, activation of leukocytes and endothelial cells
Thromboxane	Eikosanoid: activation of monocytes, differentiation of lymphocytes
nitrous oxide	Reactive oxygen species: involvement in anti-inflammatory and anti-trombotic effects
GPIbα	Adhesion molecule: binding to von Willebrand factor and to leukocytes

Abbreviations: PF4: Platelet Factor 4; PPBP, Pro-Platelet Basic Protein; CCL5: C-Chemokine Ligand 5; RANTES: Regulated upon Activation, Normal T cell Expressed and Secreted; CD40L: Cluster of Differentiation 40 Ligand; TNF: Tumor Necrosis Factor; TGF-, transforming growth factor beta; PDGF, Platelet-Derived Growth Factor; vWF: von Willebrand Factor; FGF: basic Fibroblast Growth Factor; VEGF: Vascular Endothelial Growth Factor; IGF-1, Insulin-like Growth Factor 1; MIP-1α, Macrophage Inflammatory Protein 1 alpha; MMP: Matrix MetalloProteinase; ADP: Adenosine DiPhosphate; IL-1β, Interleukin 1 beta; GPIbα, Glycoprotein Ib alpha.

**Table 2:** An overview of the most common platelet and extracellular vesicles-rich preparations and their preparation methods.

Platelet-Rich Preparation	Description and Preparation Method	Notable Points Regarding Its Use, the Main Advantages and Disadvantages of Its Use
P-PVRP/LP-PVRP	“Plasma-based” products without leukocytes and with low-density fibrin network after activation [80]. The centrifugation time is shorter than buffy-coat products, yielding plasma with a lower count of platelets compared to buffy-coat-based products (see below) [83]. Blood is collected in anti-coagulant-added test tubes and centrifuged in one or two spins: first spin to separate erythrocytes, followed by the removal of platelet-poor plasma and the buffy coat; after the second spin of the liquid, the upper two thirds are removed, and resuspension of the remainder of platelets in the lower third is performed to obtain P-PVRP [81].	Can be applied as a liquid solution (injected or topically administered on target sites) or applied in an activated gel form (by adding calcium chloride and/or bovine thrombin). The gel form is sometimes referred to as plasma/preparation rich in growth factors (PRGF) [84]. Due to anticoagulant in test tubes, the preparation can be slower with respect to PVRF preparation [81]. The gel quickly releases healing substances, and should be used after activation within an hour [27].
L-PVRP/LR-PVRP	“Buffy-coat-based” products containing leukocytes and a low-density fibrin network after activation [80]. The centrifugation time is longer than plasma-based products, obtaining a higher number of platelets along with a high density of leukocytes [83]. Prepared in one or two spins: a first spin to separate erythrocytes, followed by a second spin of supernatant plasma and the whole buffy coat. The upper two thirds are subsequently removed and the solution suspended to obtain L-PVRP.	Can be applied as a liquid solution or in activated gel form (by adding calcium chloride and/or bovine thrombin). Leukocytes have been demonstrated as beneficial in bacterial contamination prevention at wound sites and on scar tissue [31,85]. Careful patient selection is required before L-PVRP applications, as leukocytes (neutrophils) may act in a pro-inflammatory and/or catabolic way, which may negatively affect healing (see Section 2.2) [31,44,86].
L-PVRF	Products with leukocytes and a high-density fibrin network. The product is a strong fibrin matrix with complex structure with trapped platelets and leukocytes [87]. Blood sample is withdrawn into test tubes without anticoagulants; activation results in a few minutes in contact with the tube walls; the tubes are immediately centrifuged, usually at 3000 rpm for 10 min [82]. The PVRF product consists of three layers: a red thrombus in contact with the red blood corpuscle base, an acellular fibrin gel and a network of buffy columns corresponding to platelet accumulation within the fibrin clot [82]. The fibrin clot is then obtained in the middle of the tube, by pressing the clot between two layers to obtain a membranous form, ready to be used on tissues [82].	The products only exist in a strongly activated gel form, cannot be injected, but can be applied as solid material [80]. Leukocyte-rich formulation is useful in certain situations, where a very firm substance is needed [81]. The preparations involve a single-centrifugation spin and require minimal blood manipulation. Due to the firm structure of the fibrin clot and tightly trapped cells in the PRF membranes, a longer and gradual growth factor release is observed [17]. The success depends entirely on quick handling, blood collection and transfer time to a centrifuge. The membranes need to be used immediately after preparation, as they shrink due to dehydration [63,88].
P-PVRF	Products without leukocytes with a high-density fibrin network [80]. Since the cells are trapped in the fibrin clot, one of the overall advantages of fibrin matrices, the PVRF preparations are rarely prepared without leukocytes.	The preparations exist in strongly activated gel forms, which cannot be injected [80].

Abbreviations: P-PVRP, Platelet and Extracellular Vesicles-Rich Plasma; LP-PVRP, Leukocyte-Poor Platelet and Extracellular Vesicles-Rich Plasma; L-PVRP, Leukocyte, Platelet and Extracellular Vesicles-Rich Plasma; P-PVRF; Pure-Platelet and Extracellular Vesicles-Rich Fibrin or Leukocyte-Poor Fibrin; L-PVRF, Leukocyte, Platelet and Extracellular Vesicles-rich Fibrin [15,81,82,83].

**Table 3:** Studies investigating the effect of cold storage on platelet preparations.

Author, Year	Type of Storage	Temperature, Storage Duration	Study Design, Main Findings
López-García, 2016 [127]	frozen PRP	−20 °C, thawed and cooled at 4 °C prior to analysis, evaluations after 1, 2, and 4 weeks in the first month and after 3, 6 and 9 months fresh PRP for comparison was stored at 4 °C, assessed at day 0, 1, 2, 3 and 4 weeks	serum from 12 healthy donors frozen samples of PVRP in eye drop form compared to fresh PVRP samples no statistically significant differences were observed between growth factor concentration and the effects on cell proliferation and differentiation of cultured cells in fresh samples and defrosted samples after 1, 3, 4 or 9 months at −20 °C
Shiga et al., 2016 [128]	three types of storage: room temperature (RT) with shaking frozen PRP freeze-dried PVRP	one sample stored at RT with shaking was frozen and stored at −80 °C one sample freeze-dried and stored at RT measurements were done immediately after preparation, and after 2, 4 and 8 weeks of storage	blood samples from 12 healthy donors in RT samples, almost no growth factors were detected after 8 weeks, the first clear reduction of expression occurring after 2 weeks in the frozen PRP samples a significant expression of growth factors was maintained at 4 weeks, but decreased by 8 weeks for TGF-β, VEGF 2 and EGF freeze-dried PVRP maintained baseline levels of growth factors the entire 8-week duration study, with only slightly reduced levels of growth factors compared to fresh PVRP levels the researchers concluded that PVRP is best stored at −80 °C for 1 month or in a freeze-dried state for up to 6 months for preservation of crucial growth factors
Hosnuter et al., 2017 [83]	frozen PVRP	−20 °C, analyzed on the 0th, 7th and 14th day of storage	blood from 18 patients for elective plastic surgery with no chronic illnesses, divided into three groups, where the day 0 group served as the control group the study emphasizes storage in an office environment in the freezing compartment of a standard refrigerator without using a preservation agent or a special carrier container the measured growth factors (EGF, VEGF, PDGF-AB, IGF-1, TGF-β) and P-selectin levels were still present, but all growth factors significantly decreased in the frozen autologous PRP samples compared to the control group
Kim et al., 2020 [129]	cold-storage leukocyte-rich PVRP and frozen leukocyte-rich PVRP	24 °C (room temperature group) 4 °C (refrigerator group) −70 °C (deep-freezer group) in each group, four aliquots were prepared based on the time of analysis (immediately and 1, 3 and 7 days after preparation)	six healthy donors growth factor (PDGF, VEGF, FGF-B, IGF-1, TGF-β1) concentration in PRP differed significantly based on storage temperature, duration of storage and method of activation
Koga et al., 2021 [130]	freeze-dried PVRP	−20 °C for up to 1 month	five patients for sinus surgery the samples were rehydrated at surgery mixed with bone grafting materials results were assessed 4 weeks after surgery (bone regeneration in maxillary sinus floor augmentation) vertical augmented height was maintained the preparation remained safe to use in bone engineering up to 4 weeks after freeze-dried storage, with no side effects reported
DeMello et al., 2022 [131]	frozen PVRP	−20 °C for 6 months	fifteen healthy adult canine patients growth factors after freezing and long-term storage (6 months without a preservation agent) were all present in measurable quantities, surviving the long-term storage PDGF and TGF-β1 were correlated with platelet count of the final PVRP

## Data Availability

Not applicable.
